# Comparative Analyses of the Chloroplast Genomes of Patchouli Plants and Their Relatives in *Pogostemon* (Lamiaceae)

**DOI:** 10.3390/plants9111497

**Published:** 2020-11-05

**Authors:** Cai-Yun Zhang, Tong-Jian Liu, Xiao-Lu Mo, Hui-Run Huang, Gang Yao, Jian-Rong Li, Xue-Jun Ge, Hai-Fei Yan

**Affiliations:** 1 Guangdong Food and Drug Vocational College, Guangzhou 510520, China; zhangcy@gdyzy.edu.cn (C.-Y.Z.); moxl@gdyzy.edu.cn (X.-L.M.); 2 Key Laboratory of Plant Resources Conservation and Sustainable Utilization, South China Botanical Garden, Chinese Academy of Sciences, Guangzhou 510650, China; huirun.huang@scbg.ac.cn (H.-R.H.); lijianrong@scbg.ac.cn (J.-R.L.); xjge@scbg.ac.cn (X.-J.G.); yanhaifei@scbg.ac.cn (H.-F.Y.); 3 Center of Conservation Biology, Core Botanical Gardens, Chinese Academy of Sciences, Guangzhou 510650, China; 4 South China Limestone Plants Research Centre, College of Forestry and Landscape Architecture, South China Agricultural University, Guangzhou 510642, China; gyao@scau.edu.cn; 5 Center of Plant Ecology, Core Botanical Gardens, Chinese Academy of Sciences, Guangzhou 510650, China

**Keywords:** Pogostemoneae, nucleotide diversity, phylogeny, genome skimming

## Abstract

*Pogostemon* Desf., the largest genus of the tribe Pogostemoneae (Lamiaceae), consists of ca. 80 species distributed mainly from South and Southeast Asia to China. The genus contains many patchouli plants, which are of great economic importance but taxonomically difficult. Therefore, it is necessary to characterize more chloroplast (cp) genomes for infrageneric phylogeny analyses and species identification of *Pogostemon*, especially for patchouli plants. In this study, we newly generated four cp genomes for three patchouli plants (i.e., *Pogostemon plectranthoides* Desf., *P. septentrionalis* C. Y. Wu et Y. C. Huang, and two cultivars of *P. cablin* (Blanoco) Benth.). Comparison of all samples (including online available cp genomes of *P. yatabeanus* (Makino) Press and *P. stellatus* (Lour.) Kuntze) suggested that *Pogostemon* cp genomes are highly conserved in terms of genome size and gene content, with a typical quadripartite circle structure. Interspecific divergence of cp genomes has been maintained at a relatively low level, though seven divergence hotspot regions were identified by stepwise window analysis. The nucleotide diversity (*Pi*) value was correlated significantly with gap proportion (indels), but significantly negative with GC content. Our phylogenetic analyses based on 80 protein-coding genes yielded high-resolution backbone topologies for the Lamiaceae and *Pogostemon.* For the overall mean substitution rates, the synonymous (*d*_S_) and nonsynonymous (*d*_N_) substitution rate values of protein-coding genes varied approximately threefold, while the *d*_N_ values among different functional gene groups showed a wider variation range. Overall, the cp genomes of *Pogostemon* will be useful for phylogenetic reconstruction, species delimitation and identification in the future.

## 1. Introduction

*Pogostemon* Desf. is the largest genus of the tribe Pogostemoneae (Lamiaceae), consisting of about 80 species distributed mainly from South and Southeast Asia to China [1]. According to the recent infrageneric classifications [2,3], this genus has been separated into three subgenera, which are subg. *Pogostemon*, subg. *Allopogostemon* Bhatti & Ingr., and subg. *Dysophyllus* Bhatti & Ingr. The former two (*Pogostemon* sensu stricto) consist of terrestrial herb and subshrubs, whereas the latter is made up of aquatic and marshland plants [2]. However, the most recent molecular phylogenetic study showed that none of the three morphologically defined subgenera of *Pogostemon* were supported as monophyletic, and that *Pogostemon* should be classified into two subgenera (i.e., subg. *Pogostemon* and subg. *Dysophyllus*) [4].

The newly circumscribed subgenus *Pogostemon* contains 27 aromatic species [2,4], which are of great economic importance as the sources of patchouli oil (an essential oil extracted from the dried tops of aromatic *Pogostemon* plants) [5]. *Pogostemon cablin* (Blanoco) Benth. (patchouli) is a well-known aromatic species in the world, since it is one of the top 20 essential oil-yielding plants traded on the world market [5]. Like *P. cablin*, other *Pogostemon* taxa in subg. *Pogostemon*, such as *P. plectranthoides* Desf., *P. heyneanus* Benth., and *P. benghalensis* (Burm.f.) Kuntze, have also been cultivated for their essential oils (also known as patchouli oil) [2]. Nonetheless, these oils are of inferior and often variable quality [6]. Patterns of morphological variation among patchouli plants are complicated, since different methods of cultivation and diverse local climatic conditions (especially when transferred between countries) can result in substantial morphological changes in patchouli species [6]. Patchouli species have therefore been considered to be a taxonomically difficult group [2]. Thus, it is essential to generate new data to solve the species delimitation and identification issues in the group.

Chloroplast (cp) genomes have been considered as “ultra-barcode” for species/cultivar identification [7,8,9] and phylogenomic analyses [10,11,12]. Recently, obtaining hundreds to thousands of organelle loci has become routine because of rapid improvements in high-throughput sequencing (HTS) technology in the past decade [13,14]. The number of whole cp genomes has rocketed in recent years. However, there is still a lack of cp genome data for the genus *Pogostemon* (especially for patchouli species). So far, only nine cp genome sequences derived from three *Pogostemon* species are publicly available, of which only the *P. cablin* cp genomes have been well studied [9]. It is necessary to sequence and characterize more cp genomes for analyzing the infrageneric phylogeny and species identification of *Pogostemon*, especially for the economically important patchouli plants. In this study, genome-skimming data based on a high-throughput sequencing platform was generated for *P. plectranthoides*, *P. septentrionalis* C. Y. Wu & Y. C. Huang, and two cultivars of *P. cablin* (“Indonesia” and “Vietnam”). We then assembled whole chloroplast genomes for these taxa and comparatively analyzed them (including the sequences of subg. *Dysophyllus* available in GenBank). Specifically, we aimed to (1) characterize the cp genomes of *Pogostemon* and identify high-divergence hotspots, (2) characterize nucleotide substitution patterns across *Pogostemon* cp genomes, and (3) revisit the infrageneric relationships of *Pogostemon* and the backbone phylogeny of Lamiaceae based on cp genomes.

## 2. Results

### 2.1. Characteristics of Chloroplast Genomes

The detailed information of *Pogostemon* cp genomes (including publicly available data in GenBank) is listed in Table 1. We obtained 10,041,957 and 10,334,686 paired-end reads from genome skimming sequencing for *P. plectranthoides* and *P. septentrionalis*, respectively. The average sequencing depth of the cp genome was 550.4× for *P. plectranthoides* and 844.6× for *P. septentrionalis*. The length of the cp genome is 152,430 bp for *P. plectranthoides* and 152,514 bp for *P. septentrionalis*. These two cp genomes have the typical quadripartite structure, consisting of a pair of identical inverted repeats regions (IRs; 25,666–25,665 bp), separated by a large single-copy region (LSC; 83,614–83,514 bp) and a small single-copy region (SSC; 17,584–17,570 bp). The overall GC content of the cp genome is 38.3% for *P. plectranthoides* and 38.2% for *P. septentrionalis*. The newly generated cp genomes of the two *P. cablin* cultivars (“Indonesia” and “Vietnam”) were effectively identical to those of other *P. cablin* cultivars, with only one base difference [9]. We therefore arbitrarily chose the cp genome of the cultivar “Gaoyao” (GenBank no. MF445415) as a representative of *P. cablin* for further comparative analyses.

**Figure 1:**
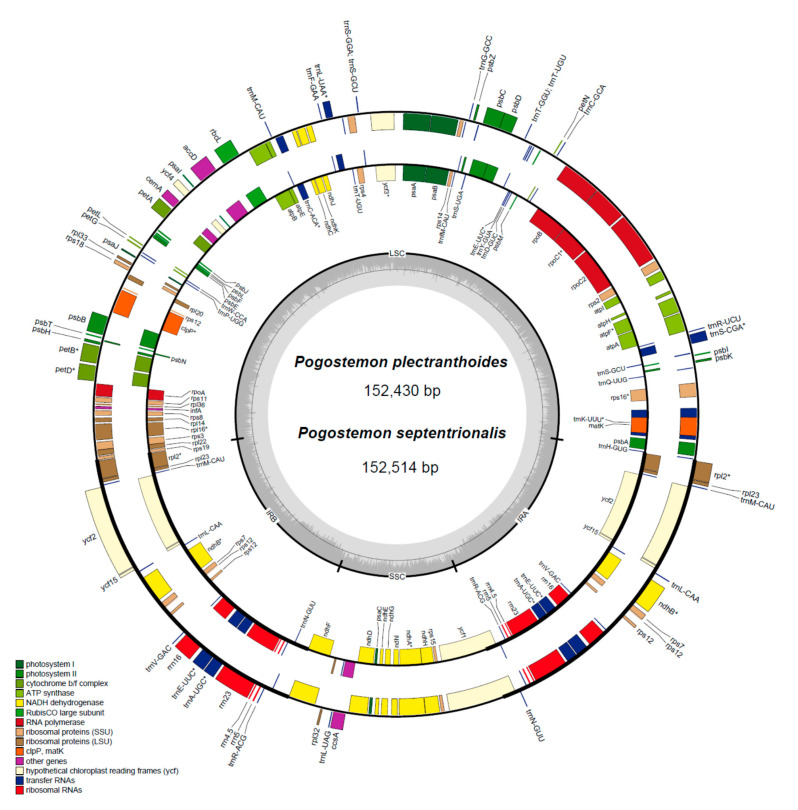
Physical map of two chloroplast genomes from *Pogostemon plectranthoides* (outer circle) and *Pogostemon septentrionalis* (inner circle). Genes inside the circle are transcribed clockwise and those outside are transcribed counterclockwise. The light gray inner circle corresponds to the AT content and the dark gray circle to the GC content. Genes belonging to different functional groups are shown in different colors. Asterisks indicate intron-containing genes.

We identified a total of 114 unique genes in the *P. plectranthoides* and *P. septentrionalis* cp genomes, including 80 protein-coding genes (PCGs), 30 tRNA genes, and four rRNA genes, consistent with the numbers identified in three other *Pogostemon* species. The gene content was conserved in these genomes, each of which has seven coding genes, seven tRNA genes, and four rRNA genes duplicated in IR regions. Besides, twelve PCGs and six tRNA genes were interrupted by introns; sixteen of these genes contained a single intron each and two genes (*clpP* and *ycf3*) possessed two introns (Figure 1).

In addition, *P. yatabeanus* (Makino) Press has the largest cp genome (152,707 bp), followed by *P. septentrionalis* (152,514 bp) (Table 1). The cp genome structures of *Pogostemon* are highly conserved, since no large genome rearrangement was detected among the five cp genomes (Figure S1). A limited variation occurred in the junction between IRs and the SSC (Figure S2). We found that the largest difference in genome size was mainly due to the indels in intergenic spacers (e.g., *trnC–petN* has 431 bp, and *trnF–ndhJ* contains 258 bp).

### 2.2. Divergence Hotspots in Chloroplast Genomes

**Figure 2:**
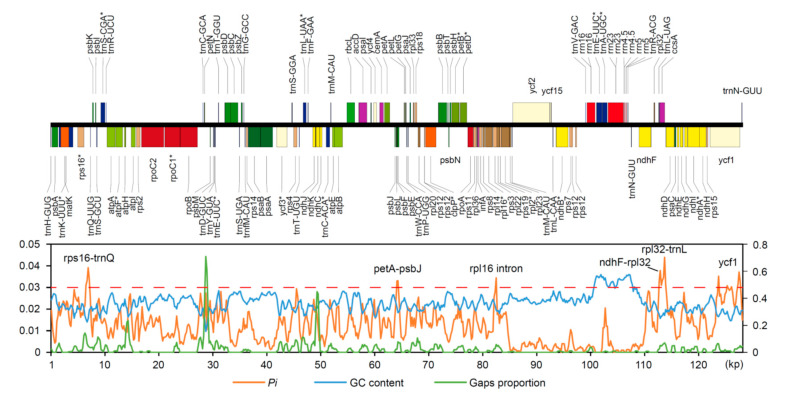
Sliding window analysis showing the nucleotide diversity (*Pi*), GC content, and gap proportion in the five *Pogostemon* chloroplast genome sequences, using 600 bp windows and a 200 bp step size.

**Figure 3:**
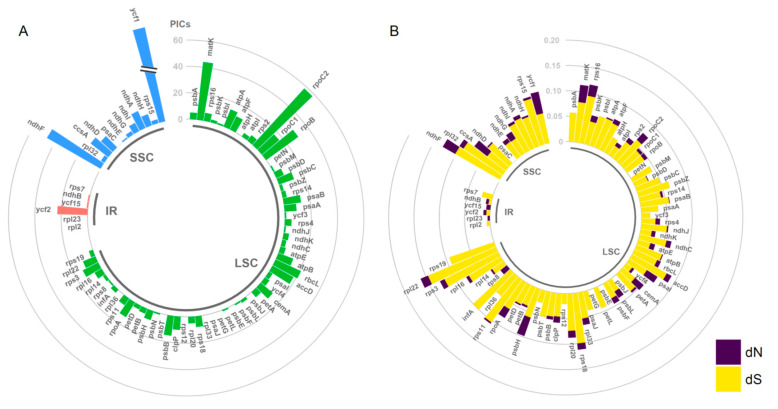
Summary of variation in phylogenetically informative characters (PICs) and substitution rates in *Pogostemon*. (**A**) Phylogenetically informative character variation in protein-coding genes in the five *Pogostemon* chloroplast genomes indicated by PICs. (**B**) Nonsynonymous (*d*_N_) and synonymous (*d*_S_) substitution rate for each protein-coding gene in the five *Pogostemon* chloroplast genomes.

Intergenic spacers (IGS) were the most highly variable regions, followed by introns, while coding regions were the most conserved regions (Figure 2). Specifically, the five most variable non-coding loci (*Pi* ˃ 0.03) identified in this study were *rps16–trnQ*, *petA–psbJ*, the *rpl16* intron, *ndhF–rpl32*, and *rpl32–trnL* (Figure 2). *Ycf1* was the only coding region with high sequence divergence (*Pi* > 0.03, Figure 2). In a comparison of PCGs using phylogenetically informative characters (PICs), *ycf1* was still the most variable region, contributing 146 PICs from an alignment length of 5583 bp (2.6%) (Figure 3A, Table S1). The other coding genes with high PICs were *rpoC2*, *ndhF*, *matK*, and *rpoB*, having 58 (1.39%), 47 (2.10%), 44 (2.86%), and 30 (0.93%) PICs, respectively (Table S1). In addition, these divergence hotspots (coding and non-coding regions) were all located in the single-copy regions (LSC and SSC).

**Figure 4:**
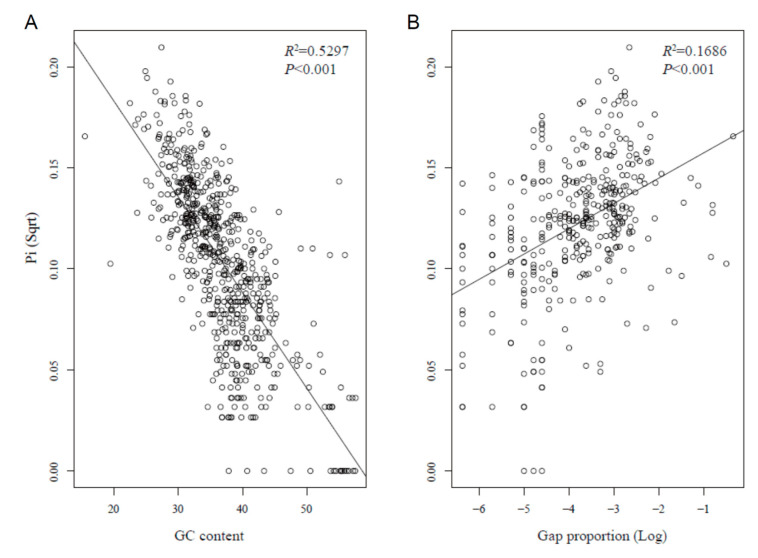
GC content and gap proportion are correlated with *Pi*. (**A**) Scatter plot showing a significant negative correlation (*p* < 0.001) between GC content and *Pi* (Sqrt). (**B**) Scatter plot showing a significant positive correlation (*p* < 0.001) between gap (indels) proportion and *Pi* (Sqrt) in the five *Pogostemon* chloroplast genomes. All three features were measured using 600 bp windows and a 200 bp step size.

We identified a significant negative correlation between *Pi* and GC content (Figure 4A), while the relationship between *Pi* and gap (indel) ratio was a positive correlation (Figure 4B), suggesting a close association between sequence variation and nucleotide composition. Our results seem to indicate that indels have a potential impact on sequence divergence in the *Pogostemon* chloroplast genome. Furthermore, large indels (see above) resulted in length polymorphism in these chloroplast genomes.

### 2.3. Phylogenetic Findings

**Figure 5:**
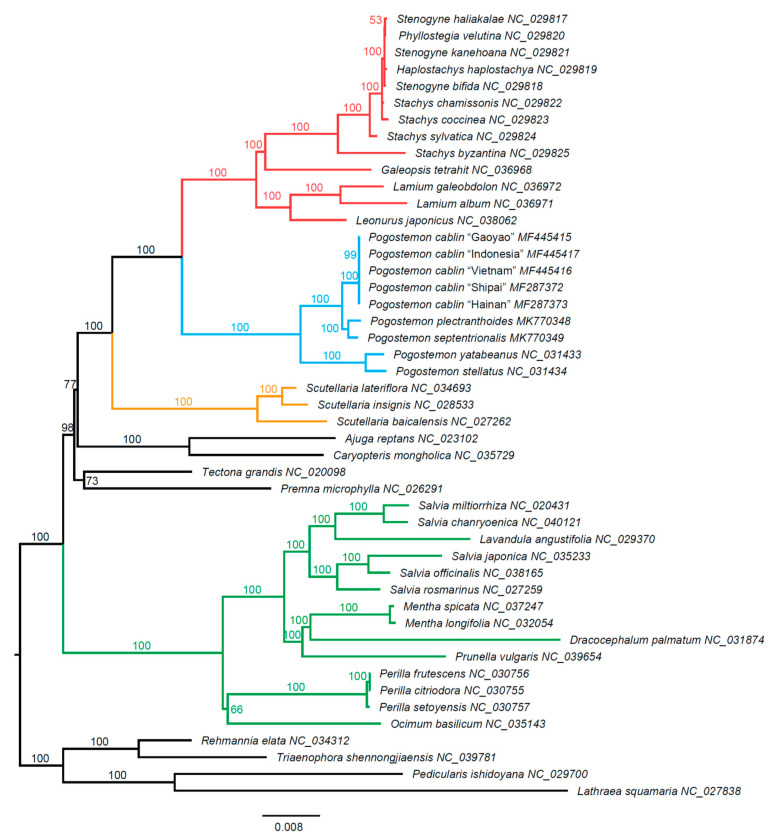
Maximum likelihood tree of 43 Lamiaceae accessions and four outgroups inferred from a concatenated codon supermatrix of 80 chloroplast protein-coding genes with a best partition scheme. Red clade: Stachydeae + *Galeopsis* + Lamieae + Leonureae, blue clade: *Pogostemon*, yellow clade: Scutellarioideae, and green clade: Nepetoideae. The group including red and blue colors is the Lamioideae. Numbers on the branches are bootstrap support values.

The chloroplast dataset comprised 80 concatenated PCGs with a total aligned length of 68,514 bp, after stripping out the sites containing more than 20% gaps. For large matrices of this type, the best practice for accommodating rate heterogeneity is grouping genes and codon positions that fitted the same best models into different partitions. Using three partitioning strategies (the best inferred gene–codon partitions, all-gene partitions, and no partitions), maximum likelihood trees were constructed and are shown in Figure 5 and Figures S3 and S4.

The topologies of the three trees were identical (Figure 5, Figures S3 and S4). We, therefore, mainly showed the ML tree based on concatenated codon matrix of 80 PCGs with a best partition scheme (Figure 5). The tree strongly supported the monophyly of the family Lamiaceae with high bootstrap support (BS) value (100; Figure 5). Ajugoideae was sister to the clade comprised of Lamioideae and Scutellarioideae with moderate support (BS = 77) in the present study. Furthermore, we recovered *Premna* and *Tectona* as a sister group with moderate support (BS = 73). In the Lamioideae, two well-supported clades were identified (Figure 5). All *Pogostemon* species formed one clade, which was subsequently divided into two subclades (i.e., subg. *Pogostemon* and subg. *Dysophyllus*), each with strong support (BS = 100). The sister group to *Pogostemon* included Stachydeae, *Galeopsis*, Lamieae, and Leonureae (Figure 5). In addition, all taxa of Nepetoideae formed a monophyletic clade (BS = 100).

### 2.4. Estimation of Evolutionary Rates among Protein-Coding Genes

Substitution rates varied considerably among the chloroplast genes across the five *Pogostemon* species (Figure 3B). For example, eighteen genes had only synonymous substitutions, whereas three contained solely nonsynonymous substitutions (Table S1). In addition, five genes had no variation representing either type of substitution event (Table S1). The mean substitution rate for all chloroplast genes was 0.008 ± 0.0095 for nonsynonymous substitution rates (*d*_N_) and 0.0467 ± 0.0303 for synonymous substitution rates (*d*_S_) (Table S1).

Among gene groups, *rpo* genes had the highest mean nonsynonymous substitution rate (0.0127 ± 0.0072), followed by *ycf* genes (0.0125 ± 0.0176), whereas the lowest nonsynonymous substitution rate occurred in *pet* genes (0.0018 ± 0.0020) (Table S2). In contrast, mean synonymous substitution rates (*d*_S_) among gene groups ranged from 0.0197 to 0.0630 (Table S2).

## 3. Discussion

### 3.1. Divergence Hotspots in Cp Genomes

Genome-wide comparisons have examined sequence divergence to decide which genes or regions to utilize for phylogenetic studies within angiosperms [15,16,17] and chloroplast biotechnology [18]. The poor phylogenetic relationships within *Pogostemon* revealed in previous studies partly resulted from the sequences without specific highly variable chloroplast regions and adequately phylogenetically informative sites [4].

In this study, we detected six highly variable loci in *Pogostemon* cp genomes, all of which are located within the eight top-ranking regions identified in 25 angiosperm lineages by Shaw et al. [17], consistent with previous findings in *Pogostemon* [9]. The phylogenetic utility of protein-coding sequences in *Pogostemon* was also evaluated by a comparison of the number of PICs. Among the protein-coding genes, *ycf1* has the highest PICs, followed by *rpoC2*, *ndhF*, and *matK*. This result is similar to the findings of Walker et al. [19]. Of the most commonly used DNA barcodes, Walker et al. [19] found that gene *rpoC2* is the top-performing gene and *matK* greatly outperformed *rbcL*. They excluded *ycf1* because of its long alignment length. However, the region *ycf1* has been recommended as a powerful barcode for use within angiosperms [20]. In this study, at least three hotspots were identified in the *ycf1* region (Figure 2), which will facilitate primer design and sequencing by the Sanger method as suggested by Dong et al. [20].

Identifying divergence hotspots has long been a central issue in chloroplast genome comparisons. In general, sequence divergence in chloroplast genomes results from several common types of variation, including substitution, insertion/deletion, duplication, and rearrangement [21]. However, few studies have taken indels or inversions into consideration when comparing sequences along chloroplast genomes [22], since a high level of indels and inversions may complicate the choice of phylogenetic markers in highly variable genomes. Given the conserved structure of cp genomes in *Pogostemon*, nucleotide diversity per site (*Pi*), gap proportion, and GC content were calculated separately in each 600 bp window. A positive relationship between nucleotide substitutions and genomic rearrangements (including indels) has been detected in previous studies of angiosperms [23]. In this study, nucleotide diversity showed a significant positive correlation with gap proportion (*p* < 0.001, Figure 4B), confirming the above finding by Jansen et al. [23]. One hypothesis to explain this is that aberrant DNA repair mechanisms accelerate substitution rates [24,25]. Moreover, we found that nucleotide diversity was negatively associated with GC content (Figure 4A). Previous studies indicated that base composition often plays an important role in chloroplast DNA sequence evolution, resulting in mutations that are spatially biased across the genome [23,26].

### 3.2. Phylogenetic Positions of Pogostemon and Its Related Taxa

In recent years, complete or nearly complete organelle genomes have become increasingly accessible, providing a powerful tool for phylogenetic studies. Our molecular phylogenetic analyses based on 80 PCGs produced a high-resolution backbone topology for the Lamiaceae. All three trees strongly supported the monophyly of the family Lamiaceae (BS = 100; Figure 5, Figures S3 and S4). Our results recognized several subfamilies as presented by Li et al. [27], i.e., the Lamioideae, Scutellarioideae, and Nepetoideae, which were fully supported by bootstrap analysis. In the Lamioideae, we confirmed the robust sister relationship between *Pogostemon* and the remaining taxa within the subfamily, a finding which supports the phylogeny of Bendiksby et al. [28] based on four chloroplast molecular markers. All *Pogostemon* species formed one group, which could be subsequently divided into two subclades with strong support (BS = 100), in agreement with the result of Yao et al. [4]. The contentious relationships among the subfamilies Ajugoideae, Viticoideae, and the genus *Tectona* were still not fully resolved, but we considerably improved the resolution of these relationships using chloroplast phylogenomics. For example, our result moderately supported the Ajugoideae as sister to the clade comprising the Lamioideae and Scutellarioideae (BS = 77). We also recovered *Premna* and *Tectona* as a sister group with moderate support (BS = 73), which agreed with the phylogenetic results of Bendiksby et al. [28] but differed from those in a recent study by Li et al. [27].

### 3.3. Substitution Rate Variation in Cp Genomes

Substitution rate heterogeneity has been observed in chloroplast genomes across different lineages of plants and among different classes of chloroplast genes [29,30]. This lineage-specific or locus-specific variation in nucleotide substitution may have played a major role in adaptive evolution in different kinds of plants. In particular, the acceleration of substitution rates for specific gene classes has been found repeatedly among many plant groups [24,31]. Drouin et al. [32] showed that the *d*_N_ and *d*_S_ rates for different genes within the cp genome are very similar, not varying by more than two- to four-fold. However, their viewpoint was based on only a few cp genes (i.e., *atpB*, *matK*, *psaA*, *psbB*, and *rbcL*), which probably resulted in an underestimation of the variation in cp genes. In the present study, the mean *d*_S_ values for different cp genes varied by about threefold (0.0197 to 0.0630), in agreement with the results of Drouin et al. [32]. However, the *d*_N_ values among different functional gene groups showed greater variation than *d*_S_ values. For example, the *d*_N_ values for *rpo* (RNA polymerase genes) and *ycf* (unknown function) genes had elevated nonsynonymous substitution rates compared with other genes, to the extent that they were seven times higher than the lowest *d*_N_ value (*pet* genes) (Table S2). The pattern of genes with accelerated *d*_N_ has been found in *Pelargonium* and *Sileneae* [33,34] and has also been reported in a comparative study on cp genomes of flowering plants [24].

The molecular mechanism underlying extremely high variation in nonsynonymous substitution rates in most plants is still elusive. It is often inferred that the variation in nonsynonymous substitution rates was more likely to result from strong coevolution with synonymous substitutions, instead of consequence from the relaxation of purifying selection [35]. However, our data did not significantly support this hypothesis, since several genes (i.e., *ycf1*, *psbH*, and *matK*) showed high nonsynonymous rates but low synonymous substitutions rates, which led to relatively higher *d*_N_/*d*_S_ (ω) values (0.7186 for *ycf1*, 0.6482 for *psbH*, and 0.4317 for *matK*) (Table S1). We inferred that positive diversifying selection acting on the above genes would be expected to result in higher nonsynonymous substitution rates.

## 4. Materials and Methods

In this study, we newly sequenced two patchouli species (*P. plectranthoides* and *P. septentrionalis*) and two cultivars of *P. cablin* (“Indonesia” and “Vietnam”) using genome-skimming technology. These species were cultivated and collected in a greenhouse at South China Botanical Garden. No specific permissions were required for the relevant locations/activities. Voucher specimens were deposited in the Herbarium of South China Botanical Garden. In order to carry out a comprehensive analysis of *Pogostemon*, publicly available chloroplast genomes sequences (*P. cablin*, *P. yatabeanus*, and *P. stellatus* (Lour.) Kuntze) from *Pogostemon* in GenBank were used in this study (Table S3).

### 4.1. DNA Extraction and Sequencing

Total genomic DNA was isolated from fresh healthy leaves using a modified cetyltrimethylammonium bromide (CTAB) method [36]. The DNA concentration was estimated using a Qubit Fluorometer with a Qubit dsDNA HS Assay Kit (ThermoFisher, Waltham, MA, USA). Short-insert (ca. 500 bp) paired-end libraries were prepared using a TruePrepTM DNA Library Prep Kit V2 for Illumina (Vazyme Biotech Co., Ltd., Nanjing, China), following the manufacturer’s protocol. Genome skimming sequencing with a 2 × 150 bp chemistry reaction system was performed on an Illumina HiSeq X Ten platform at the Beijing Genomics Institute (Shenzhen, China).

### 4.2. Assembly and Annotation

The raw reads generated from Illumina paired-end sequencing were assessed by FastQC 0.11.5 [37] and quality control was carried out using Trimmomatic 0.35 [38] to remove adapters and low-quality nucleotide bases.

Here, we used two strategies to assemble the chloroplast genome. The first was the “seed-and-extend” approach in NOVOPlasty 2.5.9 [39], which assembles adapter-free reads into rough scaffolds. The whole chloroplast genome and *rbcL* protein-coding sequence of *P. cablin* “Shipai” (GenBank no. MF287372) were used separately as seed to trigger the assembly. To assess the quality and to correct errors that occurred in the initial assembly, we mapped cleaned reads back to the resulting scaffolds in Bowtie2 v.2.3.4 [40]. After checking and refining the reads mapping graph, a consensus sequence was generated with a 90% matching threshold. The circular and ungapped consensus sequence was considered to represent the complete chloroplast genome.

In the other approach, a “de novo assembly” strategy was employed using SPAdes assembler 3.13.0 [41], with parameters “-k 21,33,55,77 --careful --cov-cutoff 10”. We used Bandage 0.8.1 [42] to visualize and manipulate the assembly graph. Each node (or contig) in the graph was used to blast against the reference cp genome (*P. cablin* “Shipai”, MF287372). Nodes without blast hits were filtered out. For unresolved loops (two or more alternative nodes with the same blast hit) in the assembly graph, we dropped the nodes with lower read coverage and retained the most reliable one. For each shared node on two intersectional paths with different blast hits, we duplicated this node as repeat sequences and placed it in two distinct paths. Finally, complete chloroplast genomes were obtained from the refined graphs.

The chloroplast genomes were annotated using Dual Organellar Genome Annotator [43]. We checked and adjusted the annotations of protein-coding genes by comparison with homologous genes from the above well-annotated reference cp genomes (*P. cablin* “Shipai”, MF287372, and *P. yatabeanus*, KP718618). The tRNA genes were annotated using ARAGORN v1.2.38 [44]. Circular genome maps were drawn on the OrganellarGenomeDRAW (OGDRAW) online service following by manual modification [45]. Finally, well-annotated cp genomes were submitted to GenBank (Table S3). Raw sequencing data in this study are archived at the National Center for Biotechnology Information under BioProject PRJNA671793.

### 4.3. Sequence and Structure Divergence

The gene order and structures were analyzed in the pairwise comparisons across the five *Pogostemon* cp genomes using Geneious R11.1.5 [46]. Three single-copy (SC) and inverted repeat (IR) region (SC–IR) junctions of all *Pogostemon* cp genomes were manually examined to investigate expansion and contraction in the IR. Multiple sequence alignments of all five *Pogostemon* cp genomes were performed in MAFFT v7 [47] under default parameters and manually adjusted in Geneious. The mVISTA program in Shuffle-LAGAN mode [48] was used to visualize the overall similarities among different cp genomes in *Pogostemon*.

To identify divergence hotspots in chloroplast genomes, sliding window analysis (the window length was 600 bp with a 200 bp step size) was employed to calculate nucleotide diversity (*Pi*), GC content, and gap proportion for each window in a *Pogostemon* cp genome alignment, with the DendroPy library and a custom Python script [49]. *Pi* indicates the average number of nucleotide differences per site between two sequences from all possible pairs in an alignment. Gap proportion shows the level of missing data caused by indels. GC content gives fundamental information about the stability of nucleotide sequences, which usually corresponds to highly conserved regions constrained by mRNA secondary structures [50]. The correlation between *Pi* and GC content or gap proportion was tested in R 3.6.1 [51]. Before fitting to a linear regression model, *Pi* and gap proportion were transformed to fit the normal distribution.

### 4.4. Phylogenetic Analyses

In order to determine the phylogenetic position of *Pogostemon* and relationships among the major groups of Lamiaceae, we collected 47 chloroplast genomes, including nine accessions from the genus *Pogostemon*, 34 other accessions in the Lamiaceae, and four outgroup accessions from the Orobanchaceae (Table S3).

Nucleotide sequences of protein-coding genes (PCGs) were extracted from the 47 annotated chloroplast genomes from the Lamiaceae. Protein-coding sequences were first translated into amino acid sequences and aligned using MAFFT v7 under the L-INS-I method. These protein alignments were then back-translated to codon alignments in PAL2NAL [52]. A matrix was generated by concatenating the codon alignments of 80 PCGs and removing sites with >20% of gaps. Given the gene and codon position partitions, the best-fitting model of nucleotide substitution for the entire dataset was determined by the corrected Akaike information criterion (AICc) in PartitionFinder 2.1.1 [53]. Maximum likelihood (ML) analyses were performed in RAxML-HPC v8.2.12 [54] based on three partitioning strategies (one partition, gene-partitioning, and best-fit partitioning schemes). All three ML analyses were evaluated with 1000 rapid bootstrap replicates.

### 4.5. Estimation of Evolutionary Rates

The nucleotide sequences of 80 protein-coding genes were extracted from five *Pogostemon* cp genomes. Each gene was codon-aligned using the L-INS-I method in MAFFT v7. Phylogenetically informative characters (PICs) were counted for each gene using a Python script. Given both codon and related protein alignments of each gene, average nonsynonymous (*d*_N_) and synonymous (*d*_S_) substitution rates were estimated using the maximum likelihood method [55] with the F3 × 4 model implemented in the codeml program in PAML v4.9 [56]. In addition, protein-coding genes were assigned to nine functional groups according to the conventional classification. The genes within a functional group were concatenated for the above tests as well. The best ML tree based on PCGs was used as a constraint tree.

## Figures and Tables

**Table 1:** Characteristics of chloroplast genomes from five species in the genus *Pogostemon*.

Genome Feature	*P. plectranthoides*	*P. septentrionalis*	*P. yatabeanus*	*P. stellatus*	*P. cablin* “Gaoyao”
Genome size	152,430	152,514	152,707	151,824	152,461
LSC length	83,514	83,614	83,791	83,012	83,553
IR length	25,666	25,665	25,674	25,644	25,662
SSC length	17,584	17,570	17,568	17,524	17,584
Total coding length	91,424	91,445	91,132	91,135	91,442
Protein-coding length	79,500	79,521	79,275	79,278	79,518
rRNA-coding length	9064	9064	9064	9064	9064
tRNA-coding length	2860	2860	2793	2793	2860
Total GC content (%)	38.3	38.2	38.2	38.2	38.2
LSC GC content (%)	36.4	36.4	36.2	36.3	36.4
IR GC content (%)	43.4	43.4	43.4	43.4	43.4
SSC GC content (%)	32.1	32.1	32	32.1	32.1
Total number of genes (total/different)	132/114	132/114	132/114	132/114	132/114
Number of duplicated genes in IR	18	18	18	18	18
Number of genes with introns (with 3 exons)	18(2)	18(2)	18(2)	18(2)	18(2)
Number of protein-coding genes (total/in IR)	80/7	80/7	80/7	80/7	80/7
Number of tRNA genes (total/in IR)	30/7	30/7	30/7	30/7	30/7
Number of rRNA genes (total/in IR)	4/4	4/4	4/4	4/4	4/4

LSC—large single-copy region; SSC—small single-copy region; IR—inverted repeats region.

## Supplementary Materials

The following are available online at https://www.mdpi.com/2223-7747/9/11/1497/s1, Figure S1: mVISTA plot showing the percent identity of plastid genomes between each of four *Pogostemon* species and the reference *Pogostemon cablin* based on pairwise global sequence alignments, Figure S2: IRa-SSC and SSC-IRb boundaries in five *Pogostemon* chloroplast genomes, Figure S3: Maximum likelihood tree of 43 Lamiaceae accessions and four outgroups inferred from a concatenated codon matrix of 80 plastid protein-coding genes with gene partitions, Figure S4: Maximum likelihood tree of 43 Lamiaceae accessions and four outgroups inferred from a concatenated codon supermatrix of 80 plastid protein-coding genes without partitions, Table S1: Number of phylogenetically informative characters (PICs), and *d*_N_, *d*_S_, and ω values for each protein-coding gene, Table S2: Mean *d*_N_, *d*_S_, and ω values for each group of protein-coding genes, Table S3: Samples used for chloroplast phylogenomic analyses in this study.
