# Supplementary material for: Comparative Analyses of the Chloroplast Genomes of Patchouli Plants and Their Relatives in Pogostemon (Lamiaceae)
Source: Plants (Basel). 2020 Nov 5;9(11):1497. doi: 10.3390/plants9111497 (PMC7694494; doi:10.3390/plants9111497)
Supplement: Supplementary file 1 [file plants-09-01497-s001.pdf]

Supplementary Materials

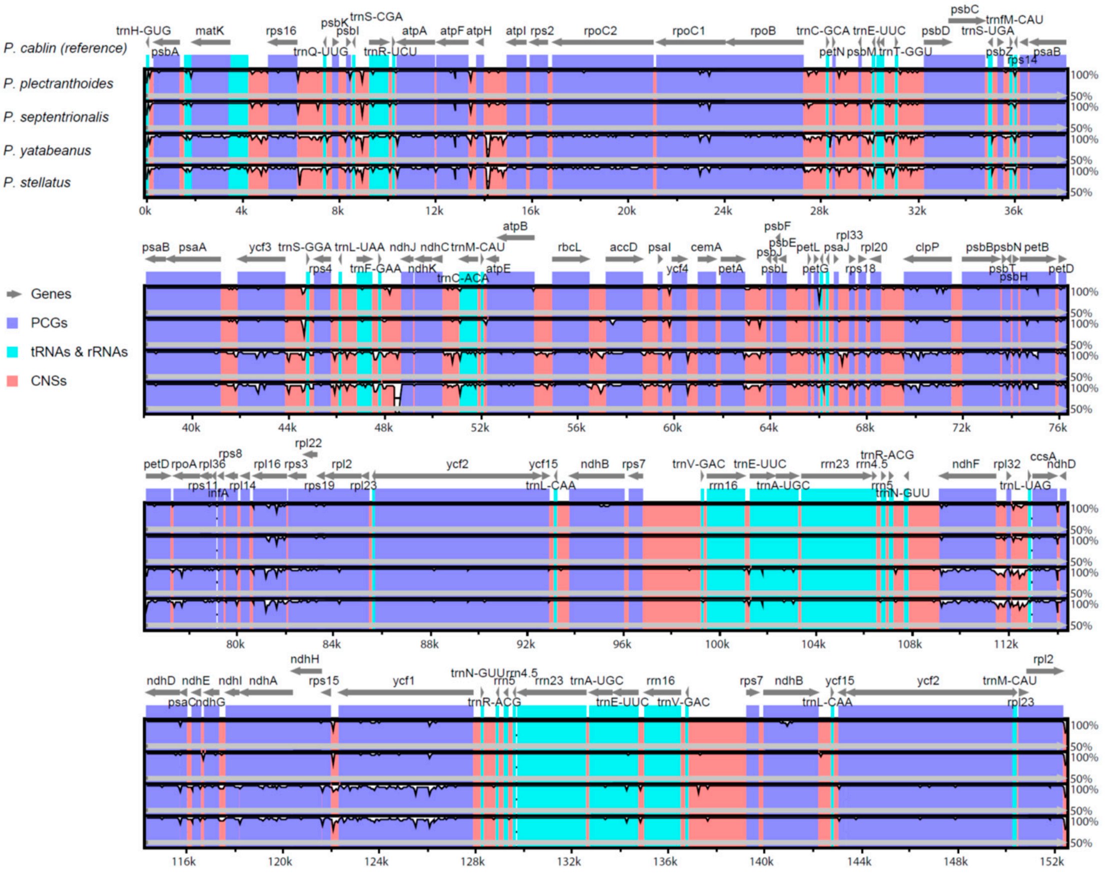

Figure S1. mVISTA plot showing the percent identity of plastid genomes between each of four *Pogostemon* species and the reference *Pogostemon cablin* based on pairwise global sequence alignments. The grey arrows at the top of the graph indicate annotated genes with their locations and orientations. Colored peaks (dark blue, protein-coding gene; light blue, tRNAs and rRNAs; pink, conserved non-coding sequences) indicate regions with at least 50% similarity calculated from 50 bp sliding windows.

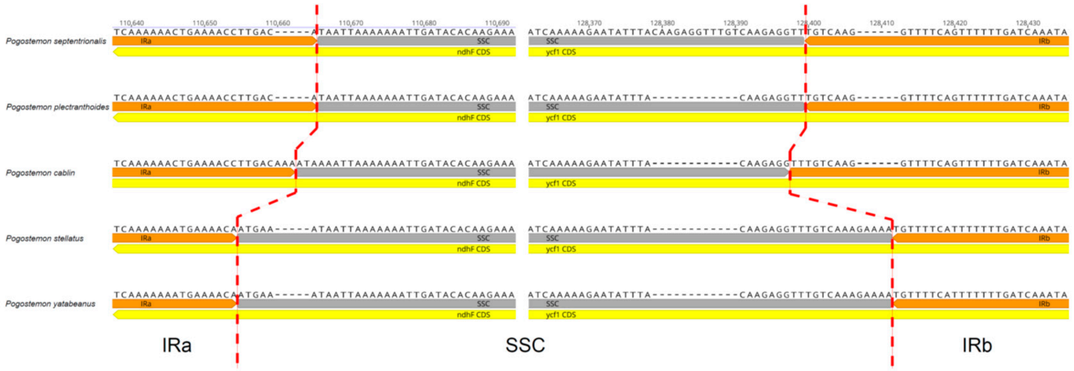

Figure S2. IRa-SSC and SSC-IRb boundaries in five *Pogostemon* chloroplast genomes.

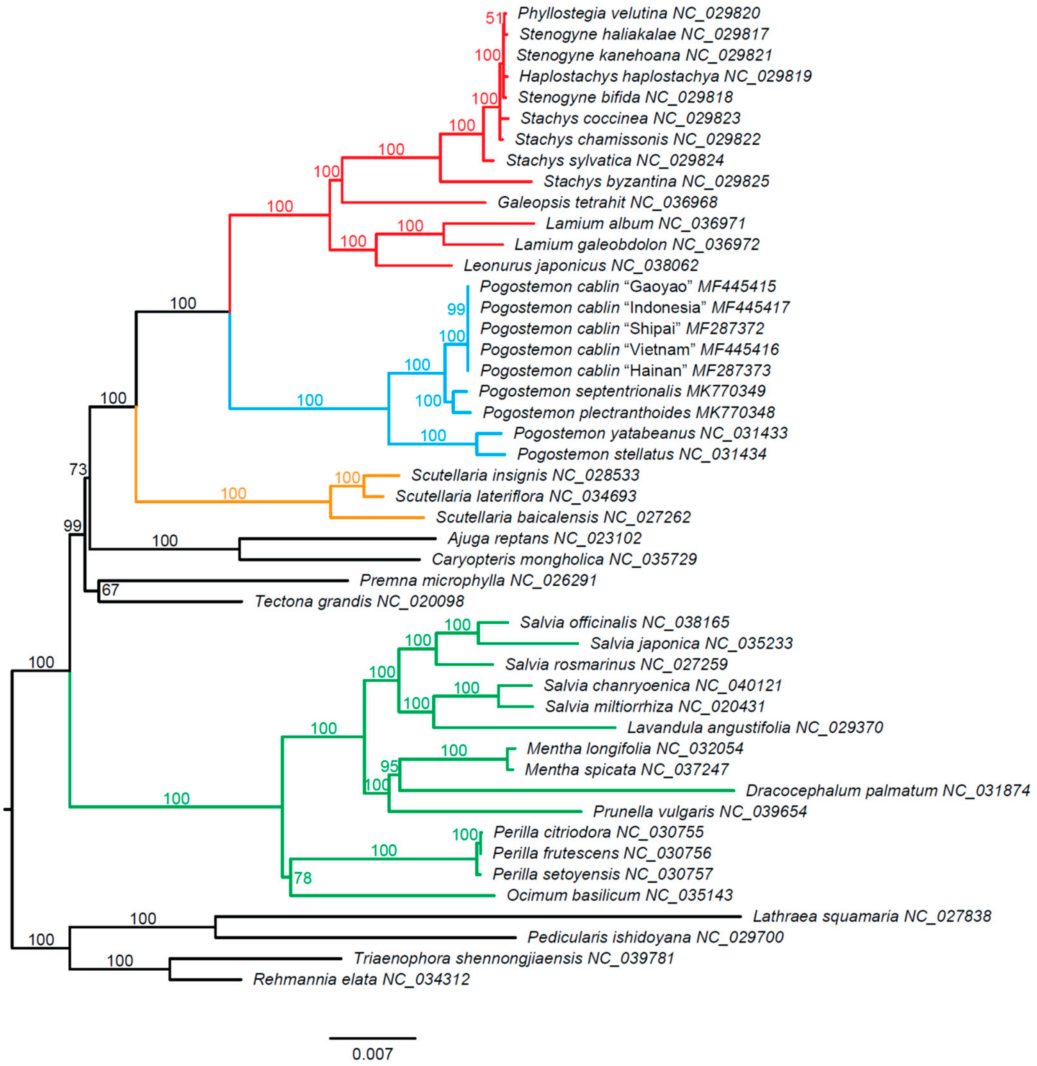

Figure S3. Maximum likelihood tree of 43 Lamiaceae accessions and four outgroups inferred from a concatenated codon matrix of 80 plastid protein-coding genes with gene partitions. Red clade: Stachydeae + *Galeopsis* + Lamieae + Leonureae, blue clade: *Pogostemon*, yellow clade: Scutellarioideae, and green clade: Nepetoideae. The group with red and blue colors is the Lamioideae. Numbers on the branches are bootstrap support values.

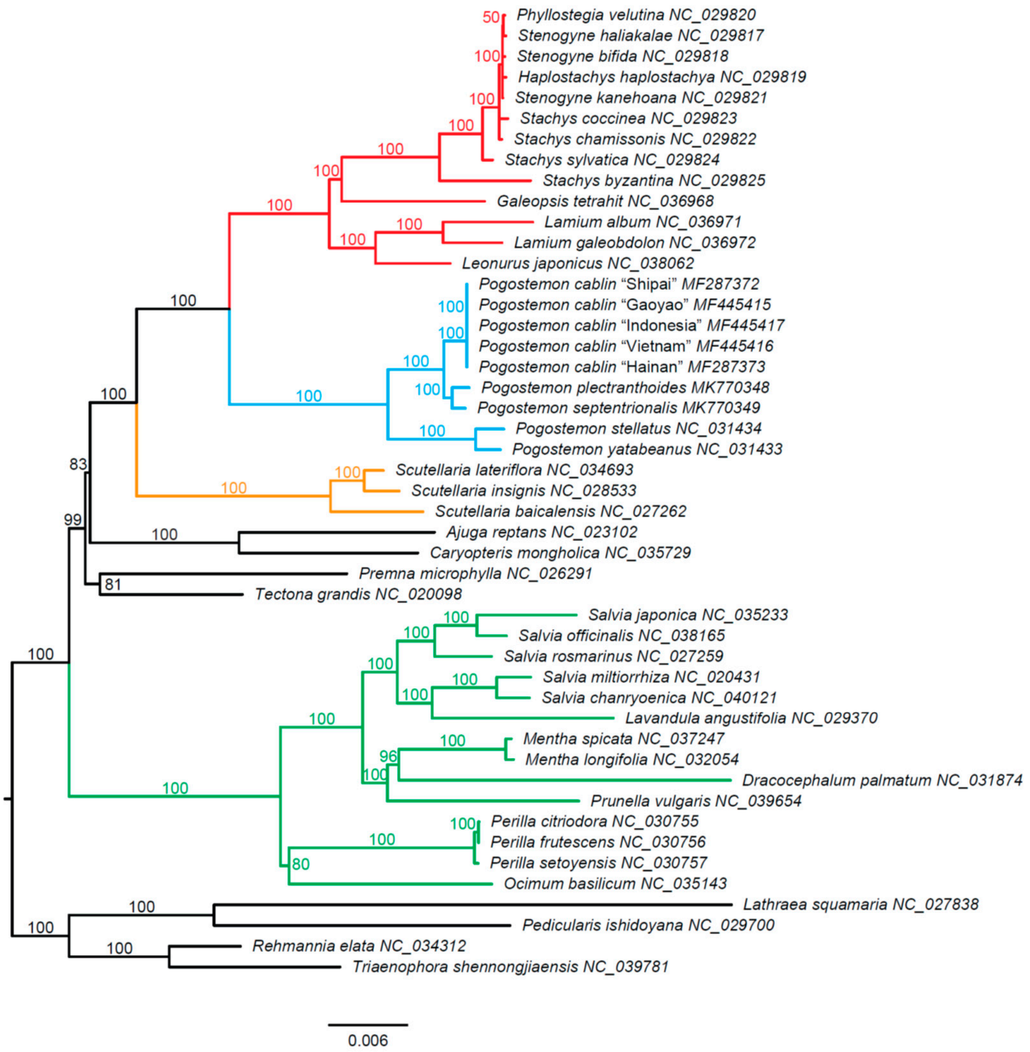

Figure S4. Maximum likelihood tree of 43 Lamiaceae accessions and four outgroups inferred from a concatenated codon supermatrix of 80 plastid protein-coding genes without partitions. Red clade: Stachydeae + *Galeopsis* + Lamieae + Leonureae, blue clade: *Pogostemon*, yellow clade: Scutellarioideae, and green clade: Nepetoideae. The group with red and blue colors is the Lamioideae. Numbers on the branches are bootstrap support values.

Table S1. Number of phylogenetically informative characters (PICs), and d_N, d_S and ω values, for each protein-coding gene.

Gene	PICs	d_N	d_S	ω
accD	18	0.0106	0.0781	0.1356
atpA	14	0.0026	0.0613	0.0417
atpB	12	0.0018	0.0540	0.0341
atpE	4	0.0097	0.0249	0.3901
atpF	11	0.0092	0.0578	0.1600
atpH	0	0	0.0334	0.0001
atpI	3	0.0075	0.0218	0.3414

ccsA	17	0.0321	0.0467	0.6867
cemA	12	0.0221	0.0316	0.7002
clpP	10	0.0121	0.0459	0.2638
infA	3	0	0.1	0.0001
matK	44	0.0341	0.0791	0.4317
ndhA	14	0.0072	0.0655	0.1098
ndhB	1	0.0054	0.0051	1.0682
ndhC	3	0.0099	0.0544	0.1825
ndhD	12	0.0026	0.0561	0.0458
ndhE	1	0.0084	0.0357	0.2351
ndhF	47	0.0283	0.1019	0.2781
ndhG	3	0.016	0.0346	0.4630
ndhH	9	0.0033	0.0621	0.0528
ndhI	7	0.0051	0.0609	0.0832
ndhJ	1	0.0026	0.051	0.0502
ndhK	4	0.0076	0.0222	0.3417
petA	5	0.0041	0.0436	0.0935
petB	4	0.0039	0.0397	0.0991
petD	3	0.0029	0.0425	0.0683
petG	0	0	0	-
petL	1	0	0.042	0.0001
petN	0	0	0	-
psaA	9	0	0.0334	0.0001
psaB	13	0.0012	0.0565	0.0205
psaC	0	0	0	-
psaI	3	0.025	0.0409	0.6101
psaJ	0	0	0.0382	0.0001
psbA	5	0	0.0571	0.0001
psbB	15	0.0061	0.047	0.1294
psbC	12	0	0.0486	0.0001
psbD	6	0.0014	0.0277	0.0490
psbE	0	0	0	-
psbF	1	0.0118	0.0333	0.3558
psbH	7	0.0384	0.0593	0.6482
psbI	1	0	0.0609	0.0001
psbJ	0	0	0	-
psbK	2	0.0124	0.0457	0.2715
psbL	2	0.0149	0.025	0.5955
psbM	1	0	0.0437	0.0001
psbN	1	0	0.0247	0.0001
psbT	0	0	0.0506	0.0001
psbZ	2	0	0.064	0.0001
rbcL	21	0.0078	0.0493	0.1577
rpl14	2	0.0035	0.0275	0.1266

rpl16	6	0.0061	0.0731	0.0836
rpl2	0	0	0.0065	0.0001
rpl20	5	0.0136	0.0736	0.1846
rpl22	10	0.0158	0.1454	0.1089
rpl23	0	0.0044	0	-
rpl32	3	0.0093	0.0937	0.0992
rpl33	0	0.0128	0.0519	0.2470
rpl36	0	0	0.0504	0.0001
rpoA	13	0.0197	0.0623	0.3158
rpoB	30	0.0073	0.0422	0.1733
rpoC1	17	0.0057	0.0425	0.1347
rpoC2	58	0.0179	0.0582	0.3071
rps11	6	0.0032	0.1037	0.0313
rps12	0	0	0.0207	0.0001
rps14	3	0.0044	0.0425	0.1028
rps15	3	0.0043	0.0943	0.0461
rps16	6	0.0224	0.0941	0.2381
rps18	8	0.0124	0.0994	0.1244
rps19	3	0	0.0931	0.0001
rps2	5	0.0096	0.0412	0.2338
rps3	13	0.0098	0.1188	0.0824
rps4	5	0.0089	0.0275	0.3236
rps7	0	0	0.0208	0.0001
rps8	1	0.0157	0	-
ycf1	146	0.0435	0.0605	0.7186
ycf15	1	0.0081	0	-
ycf2	23	0.0057	0.0072	0.7870
ycf3	1	0	0.0168	0.0001
ycf4	4	0.0050	0.0142	0.3542

Table S2. Mean d_N, d_S and ω values for each group of protein-coding genes.

Gene Group	d_N	d_S	ω
All	0.0080	0.0467	0.1704
atp	0.0051	0.0422	0.1216
ndh	0.0088	0.0500	0.1754
pet	0.0018	0.0280	0.0650
psa	0.0052	0.0338	0.1550
psb	0.0057	0.0392	0.1447
rpl	0.0073	0.0580	0.1255
rpo	0.0127	0.0513	0.2466
rps	0.0076	0.0630	0.1200
ycf	0.0125	0.0197	0.6312

Table S3. Samples used for chloroplast phylogenomic analyses in this study.

Species	Taxonomy	GenBank Accession Number
Pogostemon plectranthoides	Lamiaceae; Lamioideae; Pogostemoneae; Pogostemon	MK770348
Pogostemon septentrionalis	Lamiaceae; Lamioideae; Pogostemoneae; Pogostemon	MK770349
Pogostemon stellatus	Lamiaceae; Lamioideae; Pogostemoneae; Pogostemon	KP718620
Pogostemon yatabeanus	Lamiaceae; Lamioideae; Pogostemoneae; Pogostemon	KP718618
Pogostemon cablin "Indonesia"	Lamiaceae; Lamioideae; Pogostemoneae; Pogostemon	MF445417
Pogostemon cablin "Vietnam"	Lamiaceae; Lamioideae; Pogostemoneae; Pogostemon	MF445416
Pogostemon cablin "Gaoyao"	Lamiaceae; Lamioideae; Pogostemoneae; Pogostemon	MF445415
Pogostemon cablin "Shipai"	Lamiaceae; Lamioideae; Pogostemoneae; Pogostemon	MF287372
Pogostemon cablin "Hainan"	Lamiaceae; Lamioideae; Pogostemoneae; Pogostemon	MF287373
Premna microphylla	Lamiaceae; Lamiaceae incertae sedis; Premna	NC_026291.1
Tectona grandis	Lamiaceae; Lamiaceae incertae sedis; Tectona	NC_020098.1

Lamium album	Lamiaceae; Lamioideae; Lamieae; Lamium	NC_036971.1
Lamium galeobdolon	Lamiaceae; Lamioideae; Lamieae; Lamium	NC_036972.1
Galeopsis tetrahit	Lamiaceae; Lamioideae; Lamioideae incertae sedis; Galeopsis	NC_036968.1
Leonurus japonicus	Lamiaceae; Lamioideae; Leonureae; Leonurus	NC_038062.1
Haplostachys haplostachya	Lamiaceae; Lamioideae; Stachydeae; Haplostachys	NC_029819.1
Phyllostegia velutina	Lamiaceae; Lamioideae; Stachydeae; Phyllostegia	NC_029820.1
Stachys byzantine	Lamiaceae; Lamioideae; Stachydeae; Stachys	NC_029825.1
Stachys chamissonis	Lamiaceae; Lamioideae; Stachydeae; Stachys	NC_029822.1
Stachys coccinea	Lamiaceae; Lamioideae; Stachydeae; Stachys	NC_029823.1
Stachys sylvatica	Lamiaceae; Lamioideae; Stachydeae; Stachys	NC_029824.1
Stenogyne bifida	Lamiaceae; Lamioideae; Stachydeae; Stenogyne	NC_029818.1
Stenogyne haliakalae	Lamiaceae; Lamioideae; Stachydeae; Stenogyne	NC_029817.1
Stenogyne kanehoana	Lamiaceae; Lamioideae; Stachydeae; Stenogyne	NC_029821.1

Perilla citriodora	Lamiaceae; Nepetoideae; Elsholtzieae; Perilla	NC_030755.1
Perilla frutescens	Lamiaceae; Nepetoideae; Elsholtzieae; Perilla	NC_030756.1
Perilla setoyensis	Lamiaceae; Nepetoideae; Elsholtzieae; Perilla	NC_030757.1
Lavandula angustifolia	Lamiaceae; Nepetoideae; Lavanduleae; Lavandula	NC_029370.1
Dracocephalum palmatum	Lamiaceae; Nepetoideae; Mentheae; Dracocephalum	NC_031874.1
Mentha longifolia	Lamiaceae; Nepetoideae; Mentheae; Mentha	NC_032054.1
Mentha spicata	Lamiaceae; Nepetoideae; Mentheae; Mentha	NC_037247.1
Prunella vulgaris	Lamiaceae; Nepetoideae; Mentheae; Prunella	NC_039654.1
Salvia japonica	Lamiaceae; Nepetoideae; Mentheae; Salvia	NC_035233.1
Salvia miltiorrhiza	Lamiaceae; Nepetoideae; Mentheae; Salvia	NC_020431.1
Salvia officinalis	Lamiaceae; Nepetoideae; Mentheae; Salvia	NC_038165.1
Salvia rosmarinus	Lamiaceae; Nepetoideae; Mentheae; Salvia; Rosmarinus	NC_027259.1
Salvia chanryoenica	Lamiaceae; Nepetoideae; Mentheae; Salvia; Salvia unplaced	NC_040121.1
Ocimum basilicum	Lamiaceae; Nepetoideae; Ocimeae; Ocimum	NC_035143.1

Scutellaria baicalensis	Lamiaceae; Scutellarioideae; Scutellaria	NC_027262.1
Scutellaria insignis	Lamiaceae; Scutellarioideae; Scutellaria	NC_028533.1
Scutellaria lateriflora	Lamiaceae; Scutellarioideae; Scutellaria	NC_034693.1
Ajuga reptans	Lamiaceae; Teucrioideae; Ajuga	NC_023102.1
Caryopteris mongholica	Lamiaceae; Teucrioideae; Caryopteris	NC_035729.1
Lathraea squamaria	Orobanchaceae; Rhinantheae; Lathraea	NC_027838.1
Pedicularis ishidoyana	Orobanchaceae; Pedicularideae; Pedicularis	NC_029700.1
Rehmannia elata	Orobanchaceae; Orobanchaceae incertae sedis; Rehmannia	NC_034312.1
Triaenophora shennongjiaensis	Orobanchaceae; Orobanchaceae incertae sedis; Triaenophora	NC_039781.1
